# Octadecylamine-Grafted Graphene Oxide Helps the Dispersion of Carbon Nanotubes in Ethylene Vinyl Acetate

**DOI:** 10.3390/polym9090397

**Published:** 2017-08-27

**Authors:** Li-Chuan Jia, Zhong-Han Jiao, Ding-Xiang Yan, Zhong-Ming Li

**Affiliations:** 1 College of Polymer Science and Engineering, State Key Laboratory of Polymer Materials Engineering, Sichuan University, Chengdu 610065, China; jialichuan@stu.scu.edu.cn (L.-C.J.); jiaozhonghan@stu.scu.edu.cn (Z.-H.J.); 2 School of Aeronautics and Astronautics, Sichuan University, Chengdu 610065, China

**Keywords:** carbon nanotube, homogeneous dispersion, ethylene vinyl acetate, mechanical performance, electrical conductivity

## Abstract

In this paper, the dispersion of carbon nanotube (CNT) in ethylene vinyl acetate (EVA) is demonstrated to be significantly improved by the addition of octadecylamine (ODA)-grafted graphene oxide (GO) (GO–ODA). Compared to the CNT/EVA composite, the resultant GO–ODA/CNT/EVA (G–CNT/EVA) composite shows simultaneous increases in tensile strength, Young’s modulus and elongation at break. Notably, the elongation at break of the G–CNT/EVA composite still maintains a relatively high value of 1268% at 2.0 wt % CNT content, which is more than 1.6 times higher than that of CNT/EVA composite (783%). This should be attributed to the homogeneous dispersion of CNT as well as the strong interfacial interaction between CNT and EVA originating from the solubilization effect of GO–ODA. Additionally, the G–CNT/EVA composites exhibit superior electrical conductivity at low CNT contents but inferior value at high CNT contents, compared to that for the CNT/EVA composite, which depends on the balance of CNT dispersion and the preservation of insulating GO–ODA. Our strategy provides a new pathway to prepare high performance polymer composites with well-dispersed CNT.

## 1. Introduction

Carbon nanotube (CNT) has been regarded as a very promising nanofiller for polymer matrices to achieve high performance and multifunction, due to its extraordinary mechanical, electrical, and thermal properties [1,2,3,4,5,6,7,8]. The CNT-based polymer composites are expected to make enormous technological and commercial impacts in view of extensive applications in sensing devices [9,10], electrical shielding and heating devices [11,12,13,14,15], as well as advanced composites for space and aircrafts [16,17]. Nevertheless, the resultant performances show much lower efficiency than expected though significant efforts have been made. This is mainly attributed to the fact that CNTs easily agglomerate in polymer matrices because of the high aspect ratio and strong van der Waals interactions, which would greatly limit their utilization [18,19].

Recently, there are growing efforts on controlled surface modification of CNT to realize well dispersion through covalent or noncovalent approaches [20,21,22,23,24,25,26]. The covalent functionalization lacks economic benefits and would introduce many defects that inevitably deteriorate the intrinsic performances of CNT, whereas the noncovalent functionalization is particularly attractive because it preserves nearly all intrinsic features of CNT. For traditional noncovalent approaches, homogeneous CNT dispersion was always facilitated by surfactant and polymer wrapping based on van der Waals interactions or π–π stacking interactions. However, the surfactants may largely affect the properties of the obtained composites and the available polymers for CNT solubilization are limited [27,28]. Recently, graphene oxide (GO) was demonstrated as a high-efficient surfactant to disperse CNT due to the high solubility and adhesion of CNT onto the flat GO sheets through strong π–π stacking interaction [29,30,31,32,33]. For instance, Liao et al. reported that the incorporation of GO prominently improved the dispersion of CNTs in poly(vinyl alcohol) (PVA) and the resulted GO–CNT/PVA composite showed great improvements in the yield strength and Young’s modulus compared to those of CNT/PVA composite [31]. Fu et al. demonstrated that the tribological performance of CNT/epoxy composite was significantly enhanced by the incorporation of GO that improves the dispersion of CNT [32]. Although significant progress was achieved in developing high-performance materials, it should be noted that the GO-dispersed CNT was mainly applied in the strong polar polymer matrices, such as PVA, epoxy, and polyurethane, owing to the presence of hydrophilic functional groups (hydroxyl, epoxide, and carboxylic groups). The highly hydrophilic feature of GO makes it hardly achieve the favorable dispersion quality of CNT in weak or non-polar polymer matrices, which greatly restricts the development of such type of composites for advanced functional materials. Recently, tremendous attentions have been paid to the oleophylic modification of GO by grafting long alkyl chains to improve its dispersion in non-polar organic solvents and enhances its compatibility with weak or non-polar polymer matrices [34,35,36]. Inspired by the high efficiency of GO to disperse CNT in strong polar polymers, it is reasonable to hypothesize that long alkyl chain grafted GO should be promising and ideal to realize the efficient dispersion of CNT in weak or non-polar polymers.

In the current study, octadecylamine (ODA) grafted GO (GO–ODA) was synthesized to assist the dispersion of CNT in ethylene vinyl acetate (EVA), because CNT-based EVA composites are widely used for various applications such as packaging films, adhesives, antistatic field and electromagnetic shielding in view of their excellent flexibility, durability, and chemical resistance [37,38,39]. Significantly improved dispersion of CNT was achieved because of the strong solubilization of GO–ODA to CNT. The effect of CNT dispersion on the mechanical and electrical properties of EVA composites was investigated thoroughly. This solubilization approach for CNT with the assistance of GO–ODA is confirmed to be an effective strategy to fabricate high-performance CNT-based polymer composites.

## 2. Materials and Methods 

### 2.1. Materials

The commercially available EVA containing 28 wt % of vinyl acetate was kindly provided by Beijing Dongfang Petroleum Chemical Co. (Beijing, China). The density is 0.92 g/cm^3^ and the melt flow rate is 25 g/(10 min) (190 °C, 21.6 N). Graphene oxide (GO) was prepared from expanded graphite, which was provided by Qingdao Haida Graphite Co., Ltd. (Qingdao, China) with an expansion rate of 200 mL/g, by the modified Hummers method, as described in our previous work [40]. CNT (NC 7000 series), with average diameter of 9.5 nm, length 1.5 μm, surface area 250–300 m^2^/g, and 90% carbon purity, was supplied by Nanocyl S.A., (Sambreville, Belgium). ODA, xylene, deionized water and anhydrous ethanol were supplied by Chengdu Kelong Chemical Reagent Factory, Chengdu, China. All these chemicals were analytical reagent and used without further purification. 

### 2.2. Preparation of GO–ODA

GO–ODA was prepared by facile refluxing of GO and ODA. Specifically, 0.6 g GO was initially dispersed in 300 mL deionized water via vigorous agitation and ultrasonic treatment for 60 min. Then the resulting uniform suspension was mixed with ODA/ethanol solution (0.9 g in 90 mL) in a three-neck flask. The mixture was refluxed with intensified mechanical stirring for 24 h at 85 °C and filtrated by a PTFE membrane (0.2 μm pore size). The filtrated powder was then washed thoroughly with ethanol and filtered to remove the remaining ODA. Finally, the mixture was dried in a vacuum oven overnight at 60 °C for 48 h.

### 2.3. Preparation of GO–ODA/CNT/EVA Composites 

**Figure 1:**
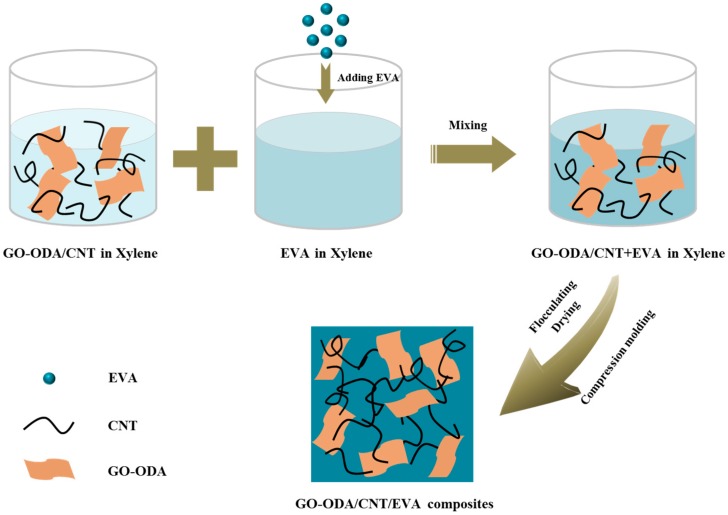
Scheme of the fabrication process of the G–CNT/EVA composites.

The schematic representation for the preparation of GO–ODA/CNT/EVA composites is illustrated in Figure 1. Firstly, GO–ODA was suspended in xylene *via* ultrasonication for 30 min, then CNT was added to GO–ODA dispersion (1:1 weight ratio) and further ultrasonicated for 60 min. Meanwhile, EVA was completely dissolved in hot xylene (75 °C) by mechanical stirring for 60 min. Subsequently, GO–ODA/CNT/xylene dispersion was poured into the EVA/xylene solution and the mixture was continuously stirred for 30 min at 75 °C. Afterwards, the mixture was recovered by flocculation with ethanol, followed by filtering and drying in a vacuum oven (60 °C) for 48 h. Finally, the dried mixture was compression molded under the pressure of 10 MPa at 160 °C, after preheating for 5 min. The resultant GO–ODA/CNT/EVA composites were marked as G–CNT/EVA composites. For comparison, the CNT/EVA composites were also prepared under the same processing conditions.

### 2.4. Characterizations

Fourier-transform infrared spectroscopy (FTIR) spectra were recorded with a 2 cm^−1^ spectral resolution on a Nicolet 6700 FTIR spectrometer (Thermo Nicolet, Waltham, MA, USA) in transmission mode. GO or GO–ODA was ground with KBr and pressed into KBr disks for FTIR measurements. X-ray diffraction (XRD) data was collected with a DX-1000 diffractometer (Dandong Fangyuan Instrument Co., Ltd., Liaoning, China) using CuKa irradiation at 40 kV in a scanning range from 2° to 30°. Water contact angle was measured by using a DSA 30 KRUSS drop shape analyzer (Kruss, Hamburg, Germany). Thermogravimetric analysis (TGA) was carried out to evaluate the thermal stability of GO and GO–ODA on a NETZSCH 209F1 (Netzsch, Bavarian, Germany), at a heating rate of 10 °C/min over 40–600 °C under nitrogen atmosphere. Scanning electron microscopy (SEM) images were taken by a field emission SEM (Inspect-F, FEI, Hillsboro, OR, USA) at an accelerating voltage of 20 kV. The specimens were cryo-fractured in liquid nitrogen and the fracture surfaces were coated with a thin layer of gold before morphological observation. The volume conductivity of the composite above 10^−6^ S/m was carried out on a four-point probe instrument (RTS-8, Guangzhou Four-Point Probe Technology Co., Ltd., Guangzhou, China) and the volume conductivity below 10^−6^ S/m was measured with a Keithley electrometer Model 4200-SCS (Keithley, Beaverton, OR, USA). Tensile test was performed on an Instron universal test instrument (Model 5576, Instron Instruments, Norwood, MA, USA) at room temperature. The loading rate was 100 mm/min and the gauge length was 20 mm. More than five samples were tested to calculate the average value and standard deviations.

## 3. Results

### 3.1. Characterization of GO–ODA

**Figure 2:**
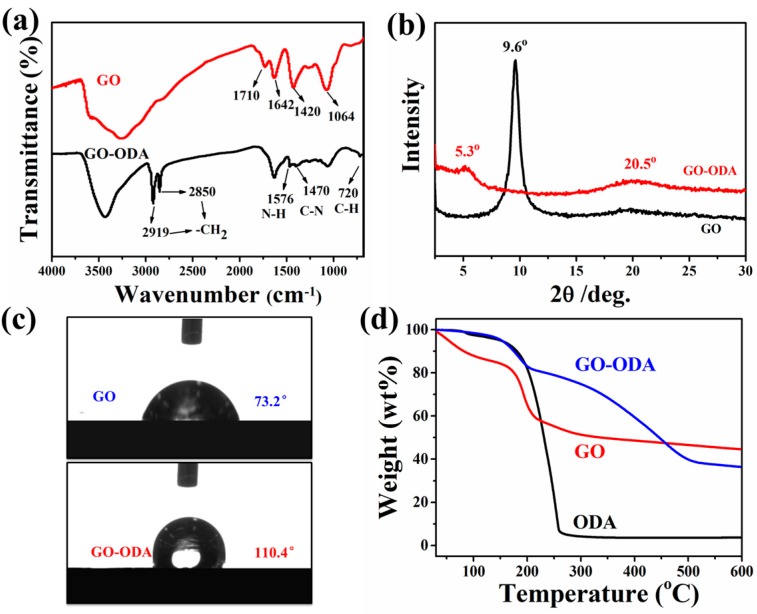
(**a**) Fourier-transform infrared spectroscopy (FTIR) spectra of graphene oxide (GO) and octadecylamine-grafted GO (GO–ODA); (**b**) X-ray diffraction (XRD) patterns of GO and GO–ODA; (**c**) Surface water contact angle of GO and GO–ODA; (**d**) TGA curves of ODA, GO and GO–ODA.

FTIR spectra were performed to determine the chemical changes that occurred during the refluxing of ODA and GO. As shown in Figure 2a, the absorption bands of GO appear at 1710, 1642, 1420, and 1064 cm^−1^, corresponding to C=O in carboxyl group, C=C in aromatic ring, C−OH stretching and C–O–C in epoxide, respectively [36]. The broad peak appearing at 3256 cm^−1^ is assigned to the hydroxyl groups. When it comes to GO–ODA, the existence of the octadecyl chain is clearly demonstrated because of the emerging peaks at 2919 cm^−1^ and 2850 cm^−1^ (–CH_2_ stretching in the octadecyl chain) as well as the peak at 720 cm^−1^ [41,42]. Furthermore, the weakened intensity of C–O–C and disappearance of C=O in carboxyl group indicate the amidation reaction between the amine functionality of ODA with the epoxy and carboxyl functionality of GO, confirming the nucleophilic substitution between ODA with GO occurred during the refluxing [36]. The new peaks at 1576 cm^−1^ (N–H bending of amide) and 1470 cm^−1^ (C–N stretch of amide) in GO–ODA also indicate the presence of amide-carbonyl bond between ODA molecule and GO. All these results affirm the intercalation and chemical reaction of ODA with GO, which is in line with the reported in the literature [36,43]. The XRD curves of GO and GO–ODA are presented in Figure 2b. Compared to diffraction peak of GO at 9.6°, the peak for GO-ODA shifts to smaller angles of 5.3°, corresponding to the increase in the intra-gallery spacing from 0.89 nm to 1.7 nm. The enlarged intra-gallery space indicates the intercalation of the octadecyl chains between the GO nanosheets, supporting the reaction of ODA and GO. The influence of functionalization on the wetting property of GO was confirmed by the contact angle measurements. The contact angle of GO–ODA increases to 110.4° in comparison with that of hydrophilic GO (73.2°), revealing that the attached hydrophobic hydrocarbon chains make the GO–ODA hydrophobic (Figure 2c). TGA was further used to analyze the amination of ODA on GO surface and calculate the grafting ratio of ODA. As shown in Figure 2d, ODA shows a rapid weight loss starting at a low temperature (120 °C), and is almost exhausted when the temperature reaches 300 °C. In case of GO, two significant weight loss stages are observed, corresponding to the volatilization of water between 40 and 100 °C and the pyrolysis of the labile oxygen containing functional groups on GO between 170 and 270 °C, respectively. Compared to GO and ODA, the larger weight loss of GO–ODA in the range of 200–500 °C is mainly attributed to the decomposition of covalently bonded ODA on GO surface, demonstrating the successful graft modification of GO with ODA. The grafting ratio of ODA is calculated to about 9.1 wt % according to the yields of residual carbon at 600 °C [36].

### 3.2. Dispersion of CNT in EVA

**Figure 3:**
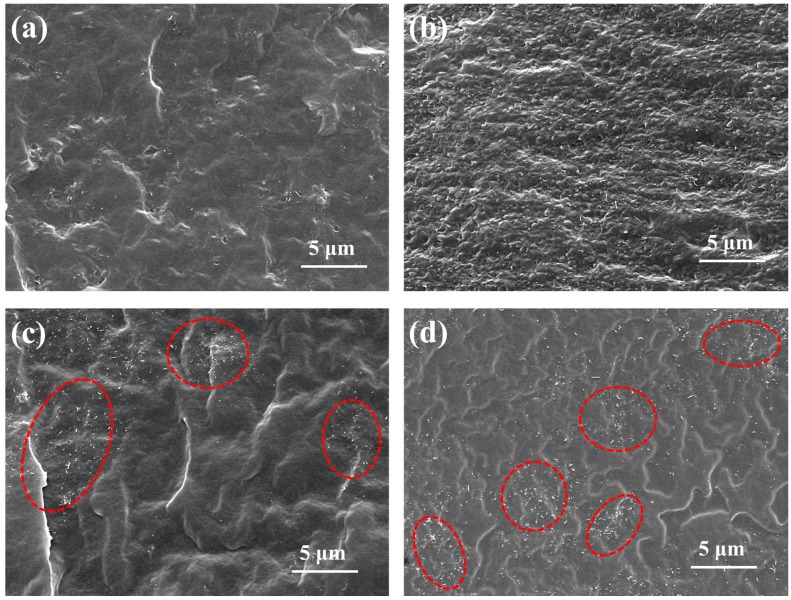
SEM images of the G–CNT/EVA composites with (**a**) 0.3 wt % and (**b**) 2.0 wt % CNT, respectively. SEM images of the CNT/EVA composites with (**c**) 0.3 wt % and (**d**) 2.0 wt % CNT, respectively. The red circles in (**c**) and (**d**) refer to the CNT agglomerations.

The dispersion of nanofillers has a direct correlation with the mechanical, electrical, and other properties of nanofiller/polymer composites. The fractured surface of the CNT-based EVA composite was observed with SEM to assess the CNT dispersion, as shown in Figure 3. For the G–CNT/EVA composites, CNTs are uniformly dispersed in EVA matrix without any visible aggregations (Figure 3a,b). While for the CNT/EVA composites, CNT agglomerations (labeled by red circle) are obviously observed (Figure 3c,d), mainly due to the intrinsic strong Van der Waals force of CNTs. These results indicate that GO–ODA plays a role of compatilizer for CNTs and EVA, which facilitates the dispersion of CNTs. Moreover, compared with the smooth fractured surface of 2.0 wt % CNT/EVA composite, the 2.0 wt % G–CNT/EVA composite shows a relatively rough fractured surface, demonstrating the excellent compatibility and strong interfacial adhesion of CNTs with EVA [36]. Based on the aforementioned analysis, the application of GO–ODA not only improves the CNT dispersion in EVA matrix but also enhances their interface adhesion, which should be attributed to the π–π stacking interaction between CNTs and GO–ODA and the interpenetration of GO–ODA chains with EVA chains. The well-dispersed CNTs in EVA matrix and high compatibility of CNTs with EVA are expected to impart the G–CNT/EVA composites with superior mechanical and electrical properties.

### 3.3. Effects of CNT Dispersion on Mechanical Properties

**Figure 4:**
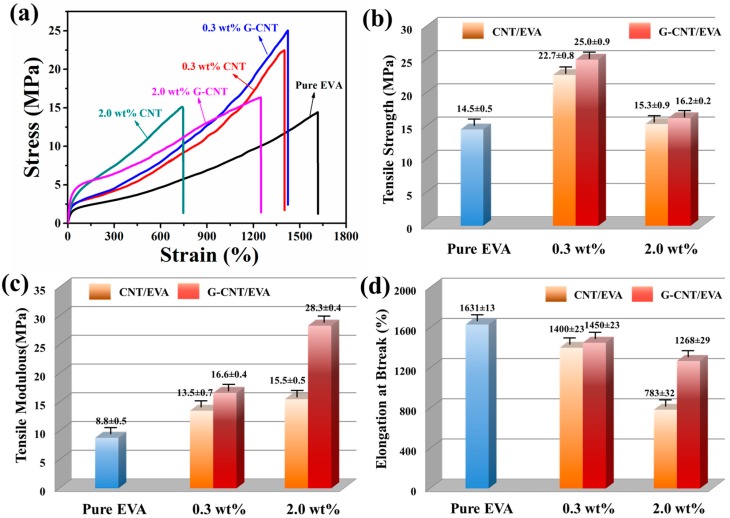
(**a**) Typical stress–strain curves of pure ethylene vinyl acetate (EVA) and its composites with various CNT loadings; (**b**) Tensile strength, (**c**) Young’s modulus, and (**d**) elongation at break of pure EVA and its composites.

Figure 4a shows typical stress–strain curves of pure EVA, G–CNT/EVA, and CNT/EVA composites. With CNT incorporation, both G–CNT/EVA and CNT/EVA composites show increased tensile strength and Young’s modulus, but decreased elongation at break, compared to those for pure EVA. The average Young’s modulus, tensile strength, and elongation at break are calculated and illustrated in Figure 4b–d, respectively. The tensile strength and Young’s modulus of the 0.3 wt % CNT/EVA composite are 22.7 and 13.5 MPa, which correspond to 56.6% and 53.4% increases from 14.5 and 8.8 MPa for pure EVA. Further increasing CNT content to 2.0 wt % in the CNT/EVA composite, the Young’s modulus increases to 15.5 MPa due to the high stiffness of CNT, while the tensile strength drops to 15.3 MPa, mainly originating from the reduced elongation at break, which should be attributed to the heavy agglomeration of CNT in EVA matrix. This phenomenon is in line with previously reported CNT reinforced polymer composites [7,25]. When it comes to G–CNT/EVA composites, the improved CNT dispersion significantly boosts up the elongation at break as compared to the CNT/EVA composite. For instance, a more than 61.9% increase in elongation at break from 783% to 1268% is achieved at 2.0 wt % CNT content. Moreover, the G–CNT/EVA composites exhibit much higher tensile strength and Young’s modulus than those of the CNT/EVA composites. The excellent mechanical performance of the G–CNT/EVA composites should be attributed to the improved dispersion of CNT and the high compatibility between CNTs and EVA molecular chain with the assist of GO–ODA, both of which facilitate effective stress transfer in the composites.

### 3.4. Effects of CNT Dispersion on Electrical Properties

**Figure 5:**
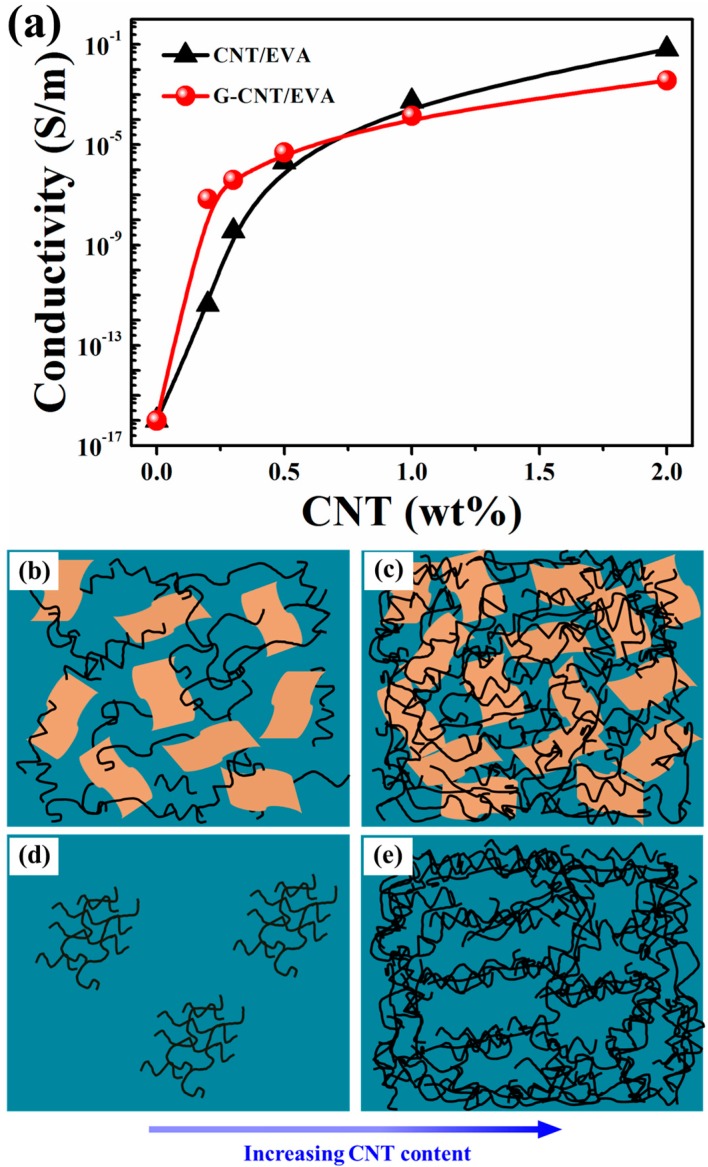
(**a**) Electrical conductivities of the G–CNT/EVA and the CNT/EVA composites. (**b**) and (**c**) The scheme of the conductive networks in the G–CNT/EVA composites at low and high CNT contents, respectively. (**d**) and (**e**) The scheme of the conductive networks in the CNT/EVA composites at low and high CNT contents, respectively.

Figure 5 shows the electrical conductivities of the G–CNT/EVA and CNT/EVA composites as a function of CNT content. Significant increases in the electrical conductivities of both the G–CNT/EVA and the CNT/EVA composites are observed with increasing CNT contents. Interestingly, the G–CNT/EVA composites exhibit superior electrical conductivities at low CNT contents, but inferior electrical conductivities at high CNT contents, compared to the CNT/EVA composites. At a very low CNT content of 0.2 wt %, the electrical conductivity of G–CNT/EVA composite already reaches 6.9 × 10^−8^ S/m, satisfying the antistatic criterion for commercial application [44]. Such value is about 4 orders of magnitude higher than that of the CNT/EVA composite (4.3 × 10^−12^ S/m). As the CNT loading rises to 0.5 wt %, the electrical conductivity of the G–CNT/EVA composites increases to 4.9 × 10^−6^ S/m, which is still higher than that of CNT/EVA composites. However, with further increasing CNT loadings to 1.0 wt % and 2.0 wt %, it can be seen that the electrical conductivities of CNT/EVA composites exceed that of the G–CNT/EVA composites. The main reasons for the interesting phenomenon can be found in the following two aspects. The first aspect is that the incorporation of GO–ODA can significantly improve CNT dispersion in EVA matrix due to π–π stacking interaction between GO–ODA and CNT. This contributes to the formation of inter-connected CNT networks. The second aspect is that the abundant presence of insulating GO–ODA between the adjacent CNTs will increase their contact resistance and thus hinder the electronic transport of CNTs in EVA. To provide visual demonstration of the conductive networks in the G–CNT/EVA and CNT/EVA composites, schematic representations are shown in Figure 5b,c and Figure 5d,e, respectively. At low CNT loading, the improved CNT dispersion contributes to the formation of effective conductive networks in the G–CNT/EVA composites (Figure 5b), while conductive networks are not formed in the CNT/EVA composites due to the agglomeration of CNTs (Figure 5d). Thus the first aspect plays a leading role in the electrical conductivity of the composites, which impart the G–CNT/EVA composites with higher electrical conductivity than that of the CNT/EVA composites. At high CNT loading, the CNT amount is enough to form inter-connected CNT networks in both the G–CNT/EVA composites (Figure 5c) and CNT/EVA composites (Figure 5e). The second aspect is dominant for the electrical conductivity of the composites and thus the G–CNT/EVA composites exhibit inferior electrical conductivity compared to that of CNT/EVA composites. A similar phenomenon was also reported for other CNT-based composites with the assistance of surfactants to improve CNT dispersion [45].

## 4. Conclusions

We demonstrate a facile and effective approach to realize the homogeneous dispersion of CNT in EVA matrix, by using GO–ODA as a compatilizer. The resultant G–CNT/EVA composites show superior mechanical properties in comparison with the CNT/EVA composites, which could be attributed to the uniform dispersion of CNT in EVA matrix and the strong interfacial interaction between CNT and EVA, originating from the solubilization effect of GO–ODA. At a very low CNT content of 0.2 wt %, the electrical conductivity of G-CNT/EVA already reaches 6.9 × 10^−8^ S/m, satisfying the antistatic criterion for commercial application. The concept of dispersing CNT by using hydrophobic GO–ODA in our work can be easily extended to other CNT-based polymer systems, which paves the way to develop such composites with excellent properties.
